# Two splice variants of the DsMEK1 mitogen-activated protein kinase kinase (MAPKK) are involved in salt stress regulation in *Dunaliella salina* in different ways

**DOI:** 10.1186/s13068-020-01786-w

**Published:** 2020-08-19

**Authors:** Ziyi Tang, Xiyue Cao, Yiping Zhang, Jia Jiang, Dairong Qiao, Hui Xu, Yi Cao

**Affiliations:** Microbiology and Metabolic Engineering Key Laboratory of Sichuan Province, Key Laboratory of Bio-Resources and Eco-Environment of Ministry of Education, College of Life Sciences, Sichuan University, Chengdu, Sichuan 610065 China

**Keywords:** *Dunaliella salina*, Alternative splicing, DsMEK1, Salt stress

## Abstract

**Background:**

*Dunaliella salina* can produce glycerol under salt stress, and this production can quickly adapt to changes in external salt concentration. Notably, glycerol is an ideal energy source. In recent years, it has been reported that the mitogen-activated protein kinase cascade pathway plays an important role in regulating salt stress, and in *Dunaliella tertiolecta* DtMAPK can regulate glycerol synthesis under salt stress. Therefore, it is highly important to study the relationship between the MAPK cascade pathway and salt stress in *D. salina* and modify it to increase the production of glycerol.

**Results:**

In our study, we identified and analysed the alternative splicing of DsMEK1 (DsMEK1-X1, DsMEK1-X2) from the unicellular green alga *D. salina*. DsMEK1-X1 and DsMEK1-X2 were both localized in the cytoplasm. qRT-PCR assays showed that DsMEK1-X2 was induced by salt stress. Overexpression of DsMEK1-X2 revealed a higher increase rate of glycerol production compared to the control and DsMEK1-X1-oe under salt stress. Under salt stress, the expression of DsGPDH2/3/5/6 increased in DsMEK1-X2-oe strains compared to the control. This finding indicated that DsMEK1-X2 was involved in the regulation of DsGPDH expression and glycerol overexpression under salt stress. Overexpression of DsMEK1-X1 increased the proline content and reduced the MDA content under salt stress, and DsMEK1-X1 was able to regulate oxidative stress; thus, we hypothesized that DsMEK1-X1 could reduce oxidative damage under salt stress. Yeast two-hybrid analysis showed that DsMEK1-X2 could interact with DsMAPKKK1/2/3/9/10/17 and DsMAPK1; however, DsMEK1-X1 interacted with neither upstream MAPKKK nor downstream MAPK. DsMEK1-X2-oe transgenic lines increased the expression of DsMAPKKK1/3/10/17 and DsMAPK1, and DsMEK1-X2-RNAi lines decreased the expression of DsMAPKKK2/10/17. DsMEK1-X1-oe transgenic lines did not exhibit increased gene expression, except for DsMAPKKK9.

**Conclusion:**

Our findings demonstrate that DsMEK1-X1 and DsMEK1-X2 can respond to salt stress by two different pathways. The DsMEK1-X1 response to salt stress reduces oxidative damage; however, the DsMAPKKK1/2/3/9/10/17-DsMEK1-X2-DsMAPK1 cascade is involved in the regulation of DsGPDH expression and thus glycerol synthesis under salt stress.

## Background

Saline soil is a severe adverse environmental factor, and more than 800 million hectares of land are affected by excess salt concentrations [1]. Adaptive responses to salt stress can be grouped into three processes: osmotic stress, ionic stress, and the detoxification response [2]. In many species, the MAPK cascade pathway is widely involved in the three processes caused by salt stress [3–5].

*Dunaliella salina* is a kind of unicellular green algae without a rigid cell wall that can survive in media containing a wide range of NaCl concentrations [6]. However, in *D. salina*, the response mechanism of salt stress has not been fully elucidated. In *Dunaliella* species, Carlos et al. found that the content of MAP kinase-like proteins increased under hyperosmotic stress in *Dunaliella viridis* [7]. DtMAPK in *Dunaliella tertiolecta* accumulates intracellular glycerol under salt stress [8]. In *D. Salina*, it is known that DsMAPK1 is differentially expressed under salt stress and temperature stress [9]. The above results suggest that there is a MAPK cascade pathway involved in salt stress regulation in *Dunaliella* species, but the regulatory mechanism of the MAPK cascade pathway in *D. salina* has not been elucidated.

The MAPK cascade pathway is minimally composed of three kinase modules: a MAP kinase (MAPK), a MAPK kinase (MAPKK, also known as MEK), and a MAPKK kinase (MAPKKK, also known as MEKK) [10]. The MAPK cascade pathway has a variety of regulatory mechanisms, including transcriptional, posttranscriptional and posttranslational mechanisms [11–13]. Alternative splicing enables the production of multiple proteins from a single gene, extending the proteomic and functional diversity in eukaryotes, which is also one of the regulatory mechanisms [14]. At present, research on alternative splicing of the plant MAPK cascade pathway is mainly focused on MAPK and MAPKKK [15–17]. In the MAPK cascade pathway, the number of MAPKK genes is the lowest [18–20]. It is puzzling, given the limited number of MAPKKs, how these kinases address the functional complexity of the MAPK cascade pathway. The inspiration for solving this problem is derived from related studies of human MEK1 and its variant MEK1b [21]. In humans, MEK1-ERK1/2 is directly involved in activating CDC25 during the G_2_/M transition to regulate mitosis [22]. Shaul et al. found that MEK1b, the alternative spliced isoform of MEK1, interacted with ERK1c, which specifically regulates Golgi fragmentation [23]. These results imply that the alternative splicing of MAPKK is responsible for distributing a variety of signal transduction pathways, which enables the MAPK cascade pathway to participate in a wider range of processes. However, the AS regulation mechanism of MAPKK was relatively unexplored, and the AS regulation mechanism of MAPKK in salt stress has not been reported.

In this report, we describe that the DsMEK1 gene undergoes alternative splicing, producing two protein splice variants, the full-size DsMEK1-X2 form and the truncated DsMEK1-X1 form that contains the disrupted protein kinase domain. DsMEK1-X2 can interact with DsMAPK1 and DsMAPKKK1/2/3/10/17. However, no interacting protein of DsMEK1-X1 has been found. The expression of DsMEK1-X2 was induced by salt stress, and DsMEK1-X1 was induced by oxidative stress. The expression of DsGPDHs was induced, and the glycerol content accumulated in the DsMEK1-X2-oe transgenic lines under salt stress. DsMEK1-X1-oe transgenic lines increased the proline content and decreased the MDA content under salt stress. These results suggest that DsMEK1 produces two different salt stress response pathways through alternative splicing: DsMEK1-X1 responds to salt stress by reducing oxidative damage, and DsMEK1-X2 responds to salt stress by regulating glycerol synthesis to maintain osmotic pressure balance.

## Results

### cDNA cloning and sequence analysis of the DsMEK1 gene

**Fig. 1:**
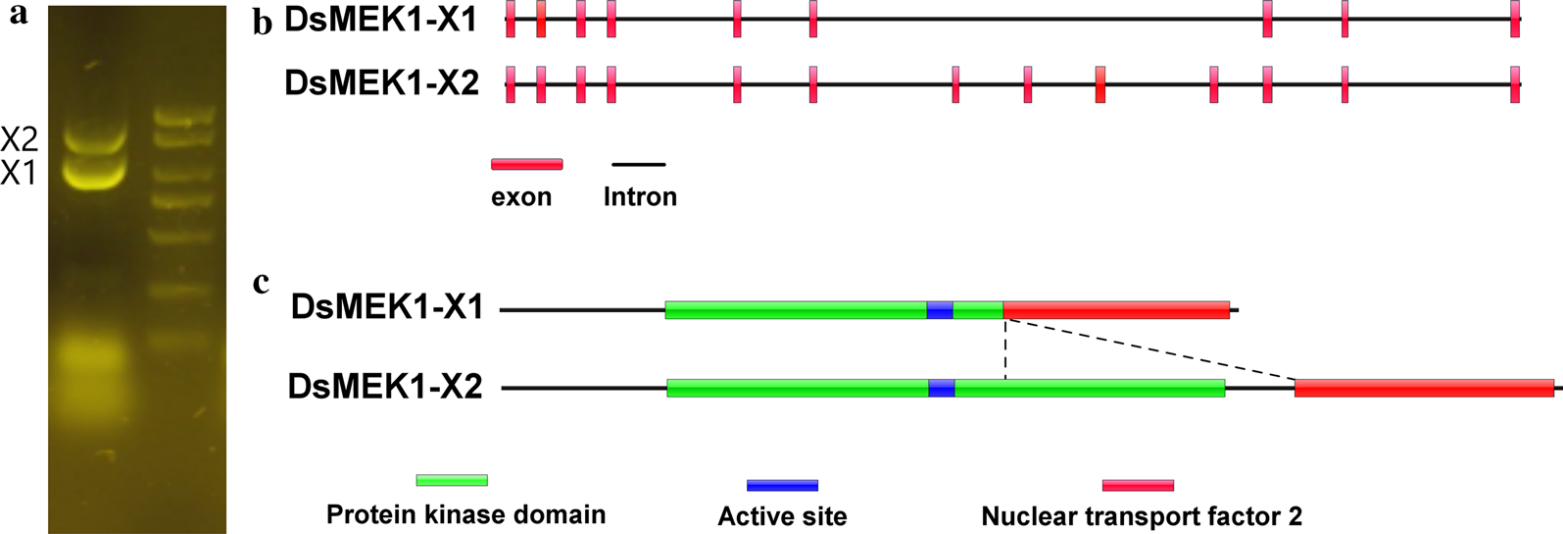
Characteristics of alternatively spliced variants of DsMEK1. **a** Detection of alternative transcripts of DsMEK1 by PCR analysis. M, 2000^+^ DNA size markers. X1 and X2 indicate the DsMEK1-X1, and DsMEK1-X2 transcripts, respectively. **b** Genomic structures of alternatively spliced variants. Black lines indicate the introns and red boxes represent exons. **c** Domain structures of protein splice variants (protein kinase domain, active site, and nuclear transport factor 2) indicated by the colour boxes. Coloured boxes mark the different regions in the proteins

To determine the sequence of the DsMEK1 cDNA isolated from the transcriptome, we conducted a RACE analysis (Additional file 1: Figure S1). According to the analysis of *D. salina* transcriptome data, there may be two alternative splices forms in DsMEK1, named DsMEK1-X1 and DsMEK1-X2, in which the length of DsMEK1-X1 is approximately 1000 bp and the length of DsMEK1-X2 is approximately 1400 bp. Two bands were separated by 2% agarose gel electrophoresis, and the sizes of the bands were in accordance with the expected sizes (Fig. 1a). These bands were purified and transformed into *Escherichia coli* DH5α and screened by colony PCR, clones containing fragments of different lengths were then sequenced, and a total of two transcript variants of DsMEK1 were obtained.

The identification of exon/intron structures for the DsMEK1-X1 and DsMEK1-X2 genes was studied by aligning full-length cDNA sequences and corresponding genomic DNA sequences. Two splicing variants were characterized; for one, 13 introns were completely spliced (DsMEK1-X2), and the other variant (DsMEK1-X1) lacked four exons (Fig. 1b). The CDS of the DsMEK1-X1 gene is 1020 bp, which encodes a 340 amino acid protein with an expected molecular weight of 37.07 kDa. The CDS of the DsMEK1-X2 gene is 1464 bp, which encodes a 488 amino acid protein with an expected molecular weight of 53.66 kDa. DsMEK1-X2 encodes the full-length protein, whereas DsMEK1-X1 encodes a truncated protein lacking a partial protein kinase domain and NTF2 domain (Fig. 1c).

**Fig. 2:**
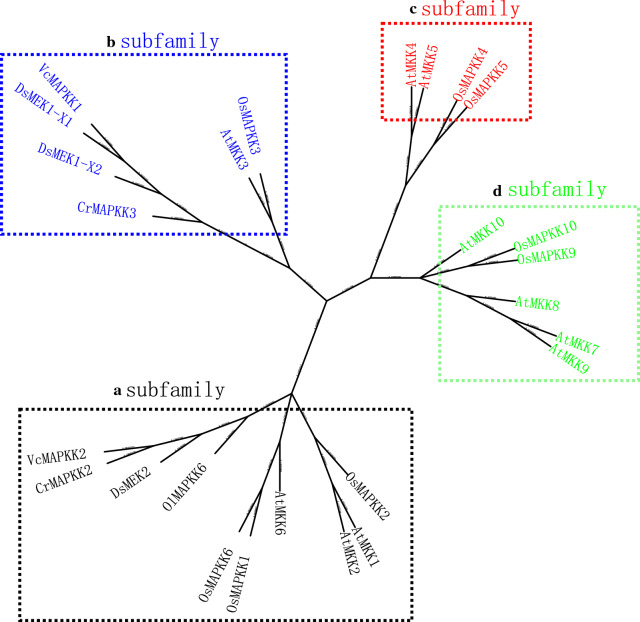
Phylogenetic analysis of DsMEK1. The evolutionary tree was constructed from the full-length MEK1 proteins from *D. salina* and all MKK proteins from *A. thaliana, O. sativa* and *C. reinhardtii*

A dendrogram was built based on the translated amino acid sequences of DsMEK1-X1, DsMEK1-X2, DsMEK2, 10 *Arabidopsis* members (AtMAPKK1-10), 8 *O. sativa* members (OsMAPKK1-8), 2 *Chlamydomonas reinhardtii* members CrMAPKK2 and CrMAPKK3, 1 *Ostreococcus lucimarinus* OlMAPKK6, and 2 *Volvox carteri* VcMAPKK1 and VcMAPKK2 (Fig. 2). The MAPKK proteins used in the analysis were divided into four groups, A–D [24]. DsMEK1-X1 and DsMEK1-X2 were categorized into subfamily B, which contains AtMKK3 and OsMKK3, suggesting that DsMEK1-X1 and DsMEK1-X2 possessed a close evolutionary relationship with AtMKK3 and OsMKK3. Previous studies revealed that AtMKK3 was induced by ABA or salt stress treatments [25], and treatment with methyl jasmonate (MeJA) or salicylic acid (SA) could induce the expression of OsMKK3 [26]. Thus, the phylogenetic tree analysis suggested that DsMEK1-X1 and DsMEK1-X2 may be involved in abiotic stress.

**Fig. 3:**
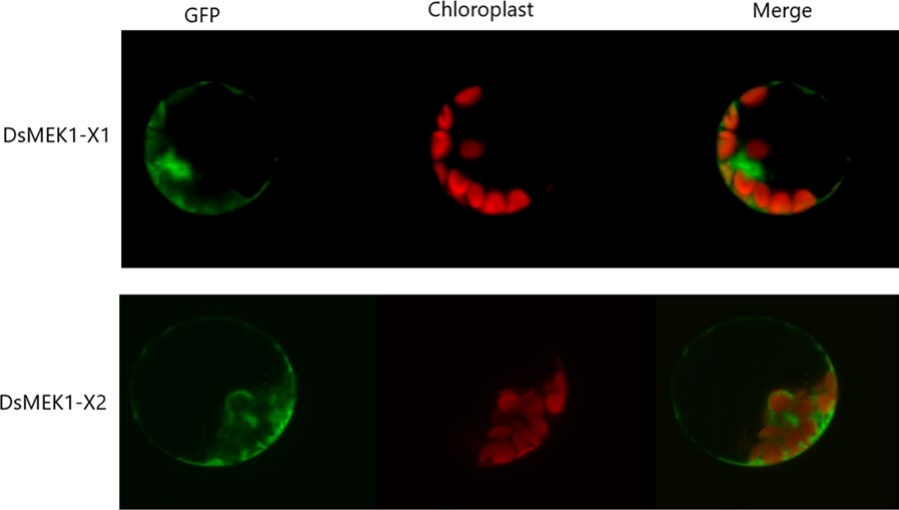
Subcellular localization of DsMEK1-X1-GFP and DsMEK1-X2-GFP fusion proteins in mesophyll protoplasts of *A. thaliana*

Database search (WoLF PSORT) with the DsMEK1 splice variant sequences pointed to the same targeting (cytoplasm). To validate the prediction, DsMEK1-X1::GFP and DsMEK1-X2::GFP were expressed in *Arabidopsis* protoplasts (Fig. 3). As expected, DsMEK1-X1::GFP and DsMEK1-X2::GFP localized to the cytoplasm. This finding suggested that splicing does not alter the localization of DsMEK1 variants in *D. salina*.

### DsMEK1 is regulated by alternative splicing

**Fig. 4:**
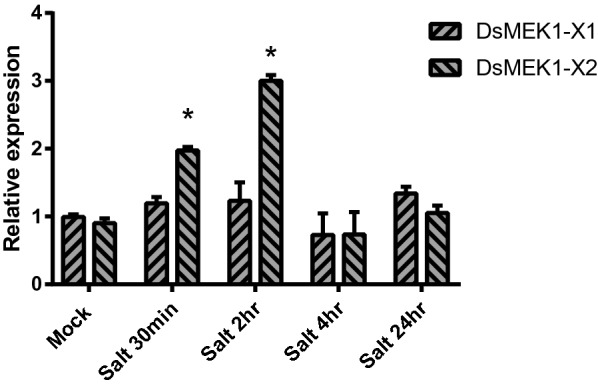
Expression profiling of DsMEK1 in *D. salina* under salt stress treatment (3.5 M NaCl). Data are presented as the means (± SE, *n* = 3). The columns with “*” had a significant difference (*p* < 0.05, fold change > 2)

For many genes, AS leads to the production of functionally different protein isoforms, which may exhibit alterations in activity, interactive partners [17], patterns of expression [27] and localization [16]. To solve the last issue, we first analysed the transcription levels of DsMEK1 isoforms under salt stress. DsMEK1-X2 was significantly upregulated along with salt treatment, while DsMEK1-X1 was nearly unaffected under salt stress (Fig. 4).

**Fig. 5:**
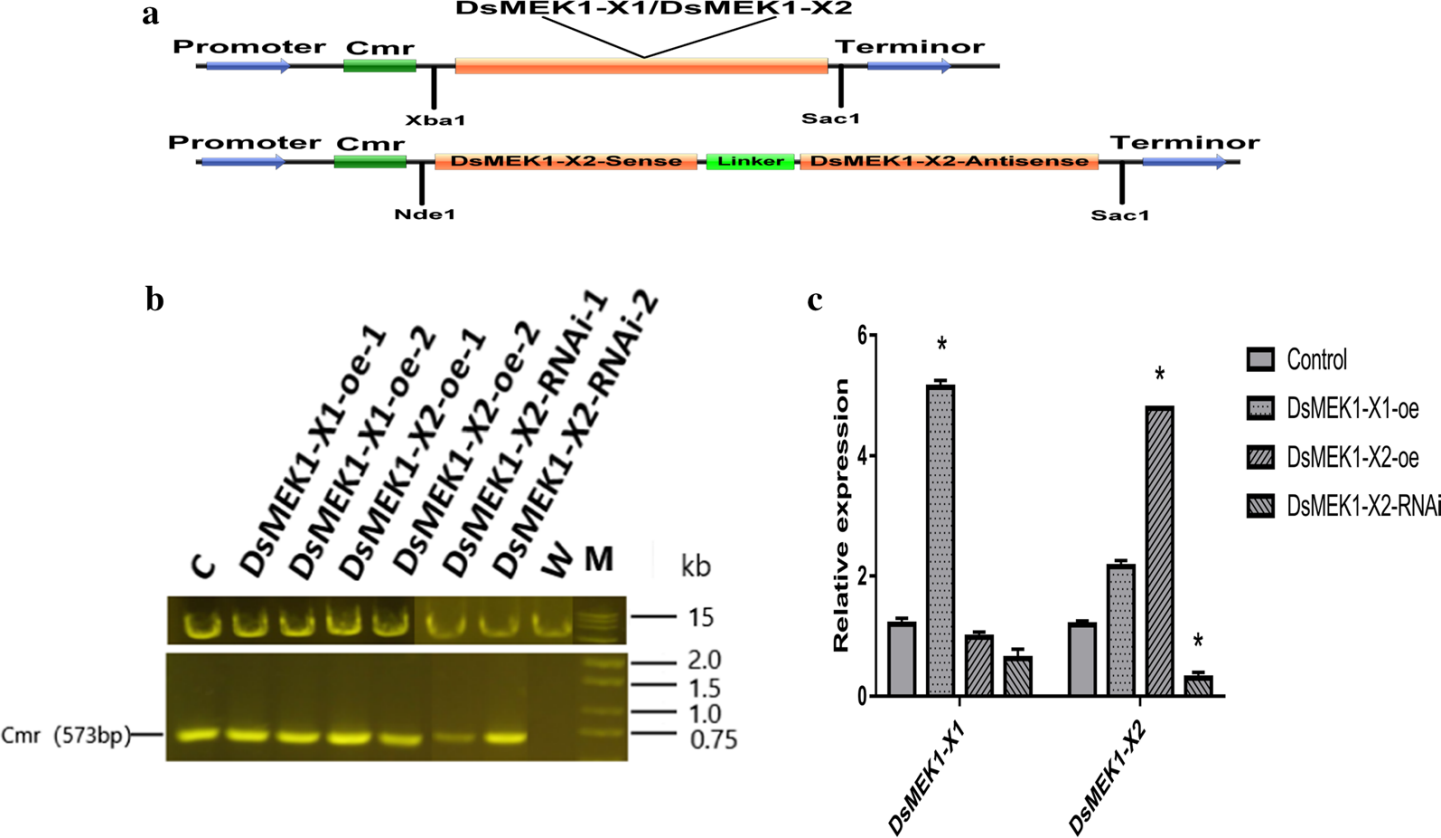
DsMEK1 transformation vector and molecular identification of DsMEK1 lines. **a** Schematic map of the DsMEK1 vector. **b** Verification of Cmr (573 bp) PCR products in the WT strain, control strain and DsMEK1 lines by agarose gel electrophoresis. **c** The expression levels of dsmek1-x1 and dsmek1-x2 in both DsMEK1-X1-oe, DsMEK1-X2-oe and DsMEK1-X2-RNAi lines, respectively. Data are presented as the means (± SE, *n* = 3). The columns with “*” had a significant difference (*p* < 0.05, fold change > 2)

Given that DsMEK1-X2 was found to be involved in the regulation of salt stress, we constructed DsMEK1-X1 and DsMEK1-X2 overexpression lines named DsMEK1-X1-oe and DsMEK1-X2-oe, respectively (Fig. 5a). Furthermore, we constructed a DsMEK1-X2 knockdown mutant, DsMEK1-X2-RNAi (Fig. 5a). In all the transformants, the Cmr gene (573 bp) was found, confirming the correct insertion of DsMEK1s in the genome of *D. salina* (Fig. 5b) [8]. qRT-PCR assays of the DsMEK1-X1 and DsMEK1-X2 genes were performed in those lines, which confirmed that the related genes were overexpressed or knockdown (Fig. 5c) [28].

**Fig. 6:**
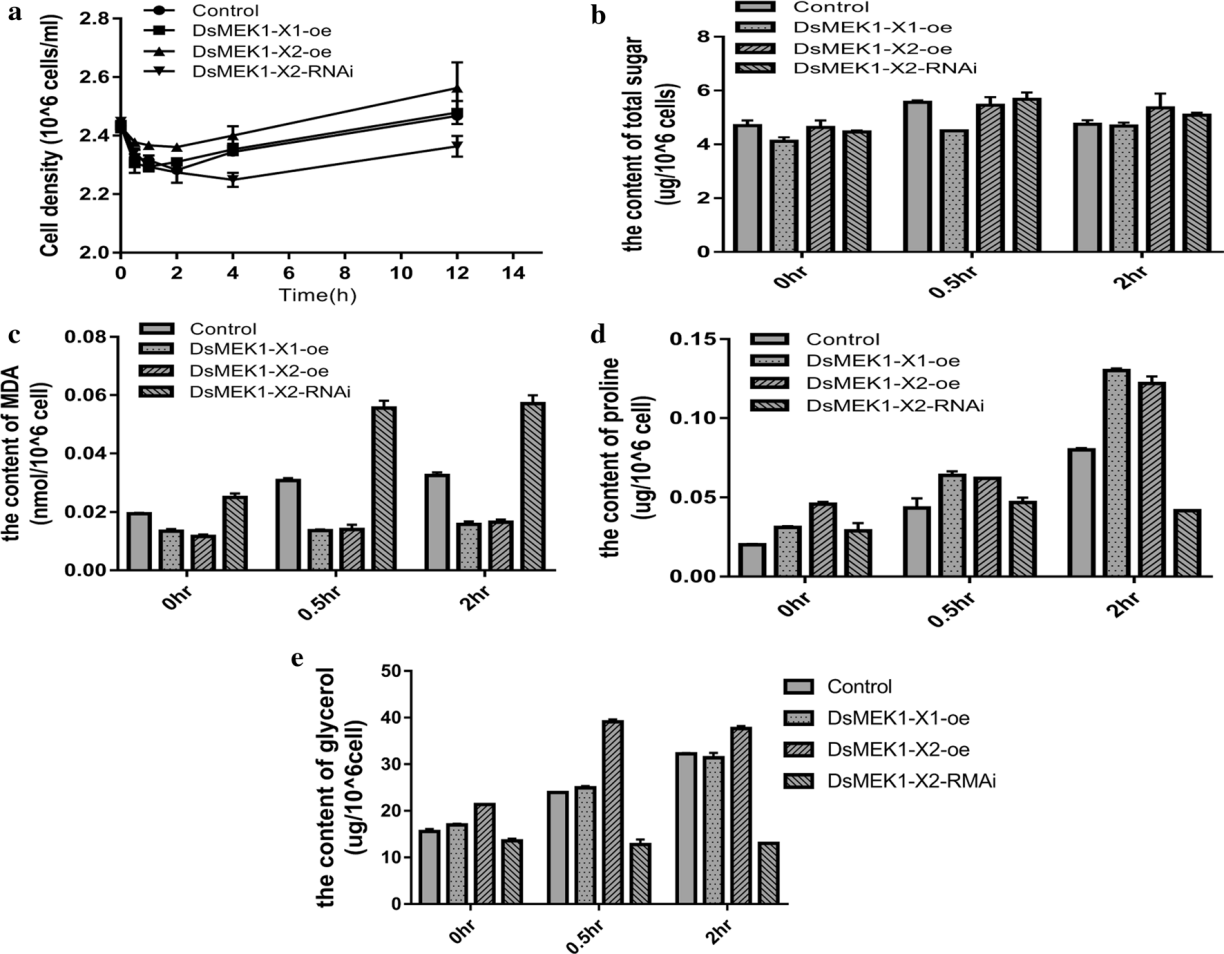
Phenotype analysis of DsMEK1-X1-oe, DsMEK1-X2-oe, and DsMEK1-X2-RNAi lines under salt stress (3.5 M NaCl). **a** The growth rates of the control strains and the DsMEK1-X1-oe, DsMEK1-X2-oe and DsMEK1-X2-RNAi lines were measured according to the cell numbers. **b** Total sugar contents, **c** MDA, **d** proline and **e** glycerol in the control strains, DsMEK1-X1-oe, DsMEK1-X2-oe and DsMEK1-X2-RNAi lines. The results are shown as the mean expression ± standard deviation (SD) of three independent experiments. Student’s *t* test, *P* < 0.05

**Fig. 7:**
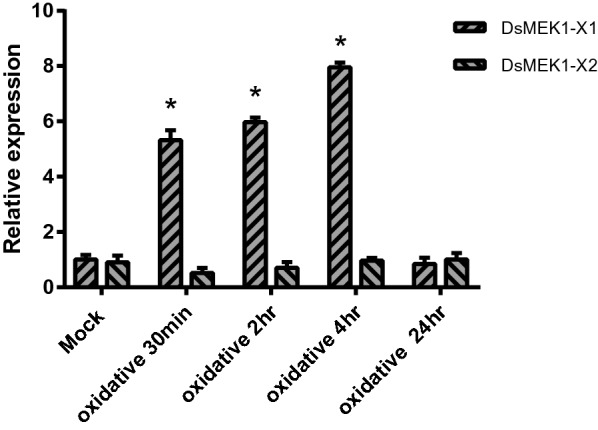
Expression profiling of DsMEK1 in *D.salina* under oxidative stress treatment (0.4 mM H_2_O_2_). Data are presented as the means (± SE, *n* = 3). The columns with “*” had a significant difference (*p* < 0.05, fold change > 2)

MDA, proline, total sugar and other compounds are often used as indicators of salt tolerance in plants. To verify the function of DsMEK1-X1 and DsMEK1-X2, we detected some physiological indexes under salt stress in *D. salina*. First, we analysed the growth curve under the salt conditions of these lines. The cell growth rate of the DsMEK1-X2-oe lines increased, and that of the DsMEK1-X1-oe lines was not significantly different from that of the control lines, while that of the DsMEK1-X2-RNAi lines was lower than that of the control lines (Fig. 6a). These results showed that DsMEK1-X2 can regulate salt stress and affect growth. The contents of total sugar content, proline content and MDA content were determined by the methods described previously. Under salt stress, the total sugar content of the DsMEK1-X1-oe, DsMEK1-X2-oe, and DsMEK1-X2-RNAi lines was not different from that of control lines (Fig. 6b). On the other hand, both overexpression strains decreased MDA content compared with the control, and DsMEK1-X2-RNAi strains increased MDA content under salt stress (Fig. 6c). Furthermore, we also detected the proline content of the DsMEK1 transformants (Fig. 6d). The proline content in DsMEK1-X1-oe and DsMEK1-X2-oe increased compared to the control. Interestingly, DsMEK1-X1-oe increased more than DsMEK1-X2-oe. The proline content in DsMEK1-X2-RNAi was less than that in the control (Fig. 6d). Previous reports have shown that MDA and proline can reduce oxidative damage [2]; therefore, we speculate that DsMEK1-X1 is involved in mitigating oxidative stress damage under salt induction. We analysed the expression levels of DsMEK1-X1 and DsMEK1-X2 under oxidative stress. As shown in Fig. 7, DsMEK1-X1 can regulate oxidative stress; however, DsMEK1-X2 does not respond to oxidative stress, which means that DsMEK1-X1 is mainly involved in antioxidant defence in response to salt stress.

*Dunaliella* species can survive in a wide range of salt concentrations because of their ability to adjust osmotic potential by changing intracellular glycerol concentrations [29]. Given that DtMAPK was found to be involved in the regulation of glycerol synthesis in *D. tertiolecta* [8], we predicted that DsMEK1 would regulate glycerol production in response to salt stress. To investigate this possibility, we analysed the glycerol content under high salinity conditions (3.5 M NaCl concentration). As expected, glycerol content in DsMEK1-X1-oe, DsMEK1-X2-oe, and the control could be enhanced significantly after salt stress. The glycerol content in DsMEK1-X1-oe had a similar increased rate compared with the control, which both increased by approximately 40% after 30 min. Interestingly, glycerol of DsMEK1-X2-oe increased almost 100% after 30 min of salt stress, which was 2.5 times that of the control and DsMEK1-X1-oe lines. Then, DsMEK1-X1-oe, DsMEK1-X2-oe and the control had similar glycerol contents after 2 h. DsMEK1-X2-RNAi strains had fewer changes in glycerol content under salt stress (Fig. 6e). These results indicate that overexpression of DsMEK1-X2 could enhance glycerol accumulation to mediate salt stress in *D. salina* cells.

**Fig. 8:**
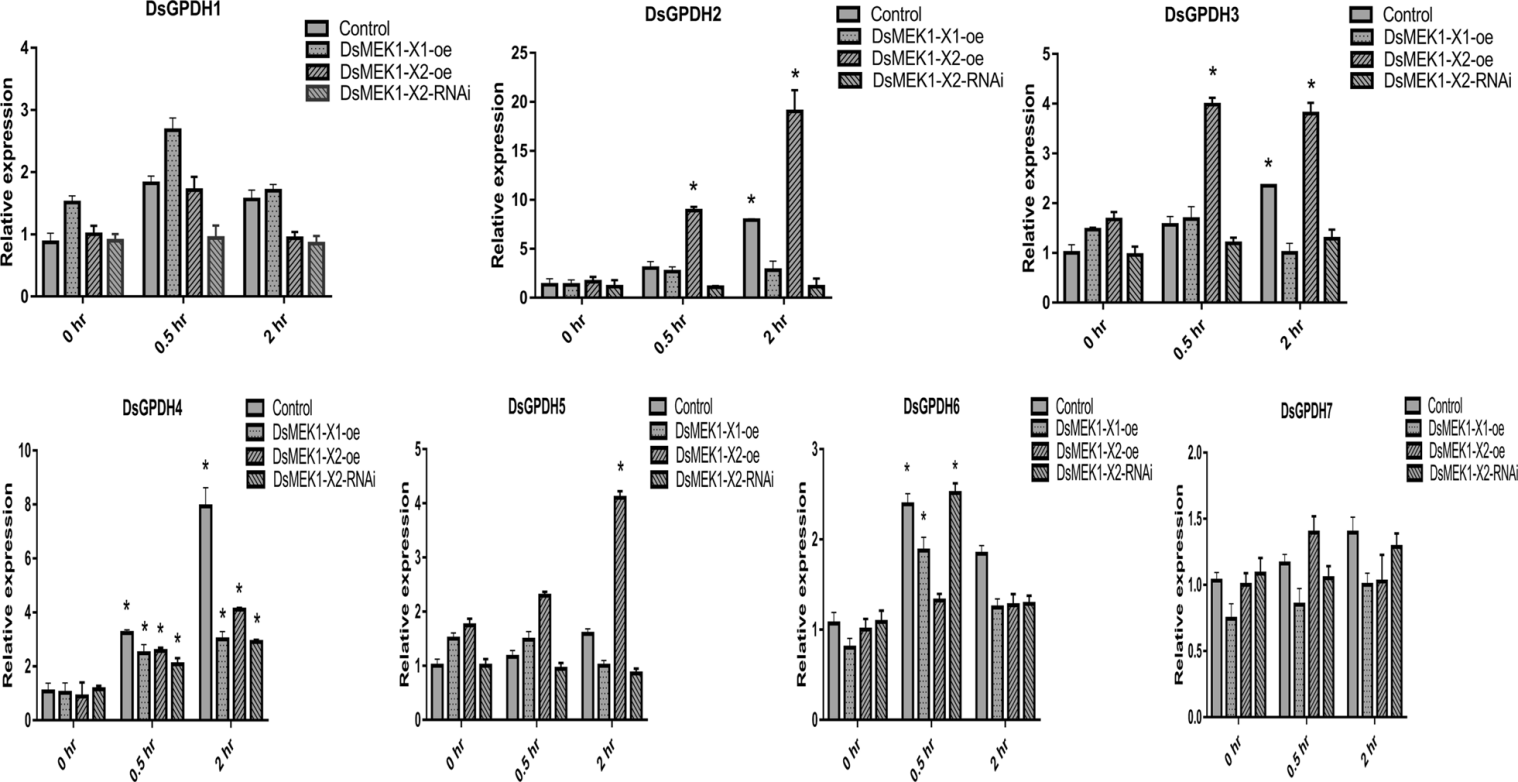
Expression levels of DsGPDH in DsMEK1-X1-oe, DsMEK1-X2-oe, and DsMEK1-X2-RNAi lines under salt stress (3.5 M NaCl), were detected by qRT-PCR assays. Data are presented as the means (± SE, *n* = 3). The columns with “*” had a significant difference (*p* < 0.05, fold change > 2)

The results reveal that DsMEK1 is involved in the regulation of glycerol synthesis under salt stress (Figs. 4 and 6). Glycerol-3-phosphate dehydrogenase (GPDH) is a rate-limiting enzyme in the glycerol synthesis pathway, and intracellular glycerol concentration functions as the counterbalancing osmolyte in *D. salina* [30]. Therefore, we further analysed the transcription levels of all DsGPDHs (DsGPDH1-7) [31], which were reported to be involved in glycerol synthesis. The expression profiles of these genes in the DsMEK1-X1-oe, DsMEK1-X2-oe, and DsMEK1-X2-RNAi lines and the control were analysed by qRT-PCR under salt stress (Fig. 8). There was no difference in DsGPDH1/7 genes between the control strains and the DsMEK1-X1-oe, DsMEK1-X2-oe, and DsMEK1-X2-RNAi lines under salt stress. The expression of DsGPDH4 was upregulated in all strains under salt stress. These results mean that DsGPDH1/4/7 was not regulated by DsMEK1 under salt stress. DsGPDH2/3/5 genes were upregulated in the DsMEK1-X2-oe strains compared to the control under salt stress. The expression of DsGPDH2 increased almost 4 times, the expression of DsGPDH3 was upregulated approximately 2.5 times and the expression of DsGPDH5 increased approximately 2 times in the DsMEK1-X2-oe strain compared to the control. DsMEK1-X2 can positively regulate DsGPDH2/3/5 expression and thus glycerol synthesis under salt stress. Furthermore, DsGPDH6 was upregulated in the DsMEK1-X2-RNAi strains and control lines, and DsGPDH6 was speculated to be negatively regulated by DsMEK1-X2 under salt stress. Based on the data analysis, it was confirmed that DsMEK1-X2 can mediate DsGPDH2/3/5/6 and is essential for the regulation of glycerol synthesis under salt stress.

### Interaction of DsMEK1 splice variants with their upstream and downstream interactors

**Fig. 9:**
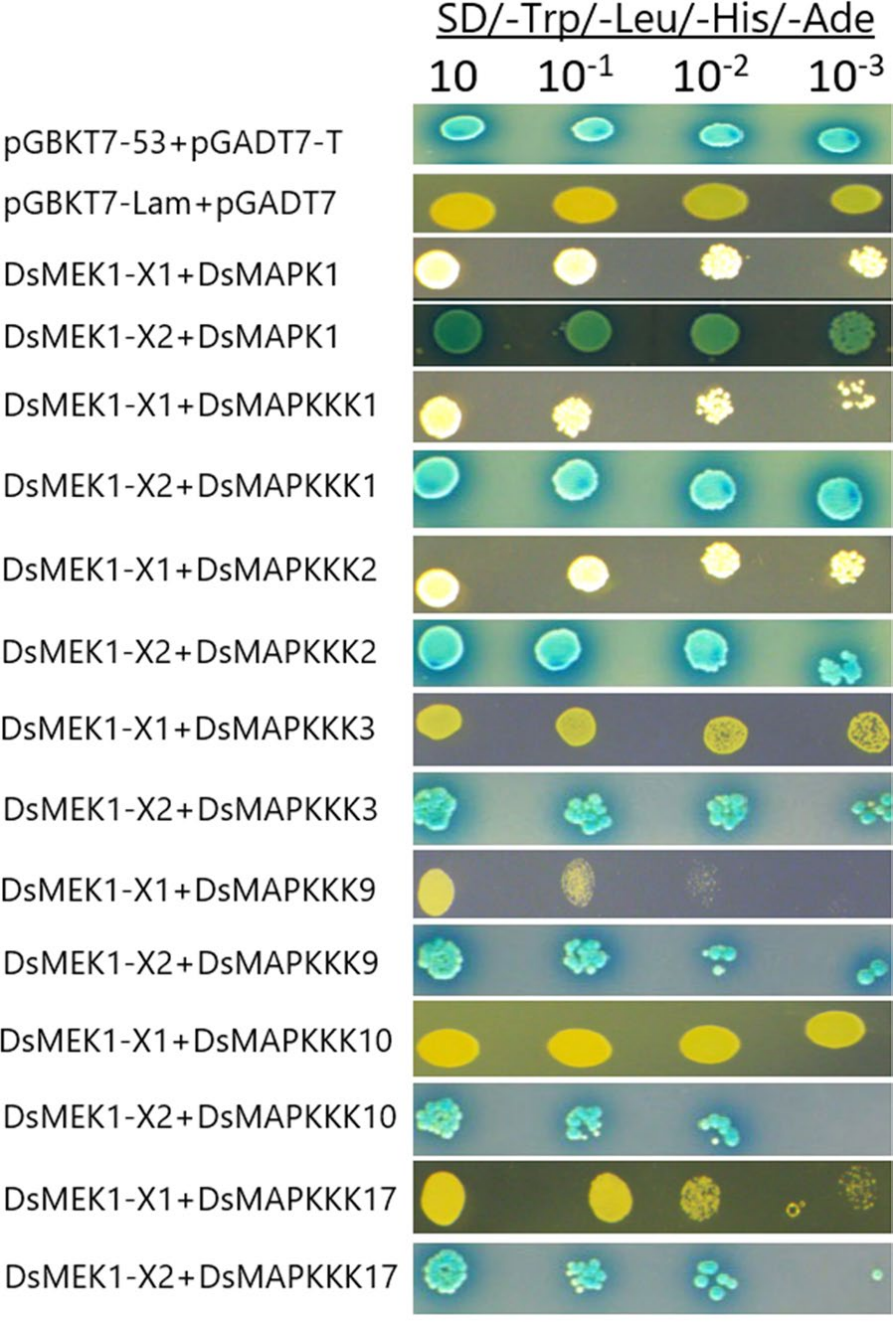
Interactions of DsMEK1 splice variants with six MAPKKKs and one MAPK in yeast two-hybrid assay. We confirm that our bait does not autonomously activate the reporter genes in Y2H Gold in the absence of a prey protein

Alternative splicing could provide a selective advantage for choosing upstream and downstream regulators [17, 32]. Therefore, we investigated the effect of AS on the regulation of the variants by performing a Y2H assay. The Y2H results showed that DsMAPK1 [9] and DsMAPKKK1/2/3/9/10/17 interacted with DsMEK1-X2 but not with DsMEK1-X1 (Fig. 9).

**Fig. 10:**
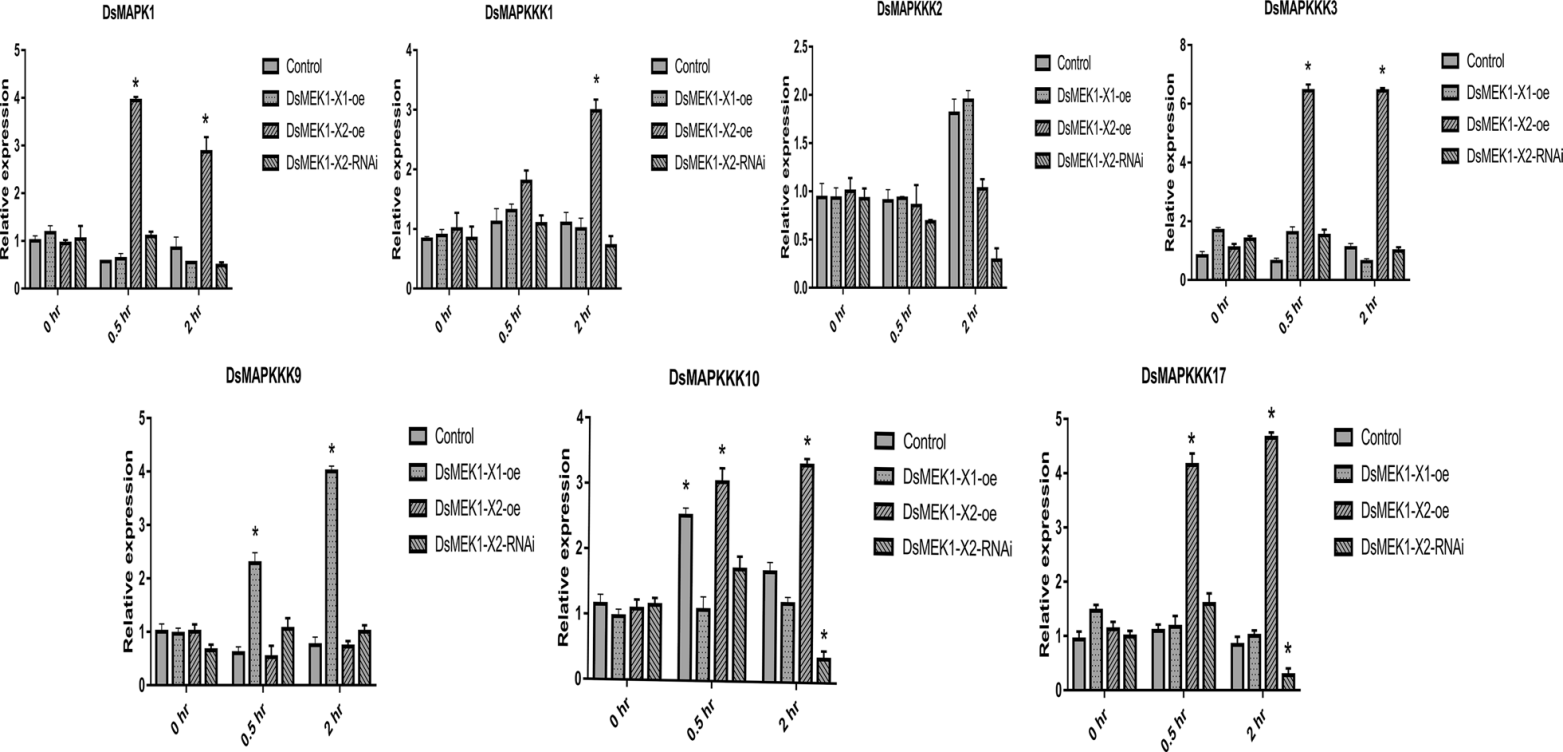
Expression of DsMAPK1 and DsMAPKKK1/2/3/9/10/17 in DsMEK1-X1-oe, DsMEK1-X2-oe and DsMEK1-X2-RNAi lines under salt stress (3.5 M NaCl), were detected by qRT-PCR assays. Data are presented as the means (± SE, *n* = 3). The columns with “*” had a significant difference (*p* < 0.05, fold change > 2). Data are presented as the means (± SE, n = 3). The columns with “*” had a significant difference (*p* < 0.05, fold change > 2)

To further investigate the function of DsMEK1, we analysed the expression of DsMAPKKK1/2/3/9/10/17 and DsMAPK1 in the control, DsMEK1-X1-oe, DsMEK1-X2-oe and DsMEK1-X2-RNAi lines under salt stress (Fig. 10). Notably, DsMAPK1 and DsMAPKKK1/3/10/17 were upregulated in DsMEK1-X2-oe lines under salt stress, and DsMAPKKK2/10/17 were downregulated in DsMEK1-X2-RNAi lines under salt stress. Combined with the results of Y2H, we confirmed that DsMAPKKK1/3/10/17-DsMEK1-X2-DsMAPK1 positively regulates salt stress and that DsMAPKKK2-DsMEK1-X2 negatively regulates salt stress.

## Discussion

Alternative splicing is involved in a variety of signal transduction processes [32, 33]. The MAPK cascade pathway interacts and transmits signals from the upstream receptor to downstream functional targets by phosphorylation, thereby causing the cells to produce a series of stress-resistant reactions and playing a pivotal role in development or stress response. In humans, MEK1 regulates the MAPK cascade pathway by selective splicing [21–23]. However, aside from this article, there are no other reports about MAPKK alternative variants. In the present study, we searched the *D. salina* transcriptome database and identified two alternative splicing genes, DsMEK1-X1 and DsMEK1-X2. The lengths of DsMEK1-X1 and DsMEK1-X2 were 340 and 488 amino acids, respectively. The above results imply that MAPKKK alternative splicing is a useful means of regulating the MAPK cascade pathway.

**Fig. 11:**
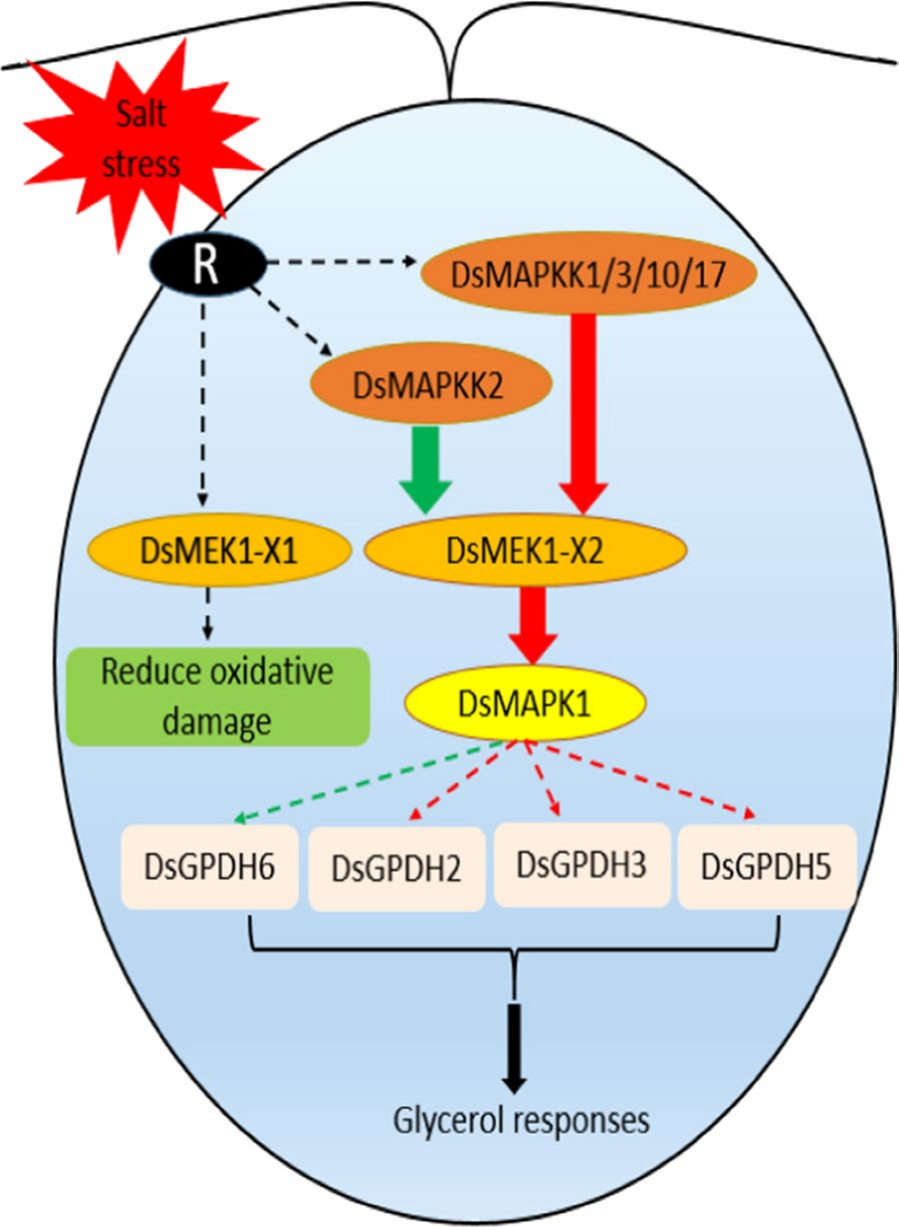
Hypothetical model depicting the functional role of DsMEK1 splice variants and upstream and downstream targets in salt stress signalling in *D. salina*. Solid arrows depict known pathways, while dashed arrows indicate proposed or unknown pathways. Red lines indicate positively regulated, green lines suggest negative regulation

Alternative splicing can lead to changes in the physiological function of organisms to help organisms deal with a variety of stresses [34]. In our study, there were differences between DsMEK1-X1 and DsMEK1-X2 in the response to salt stress. DsMEK1-X2 led to the rapid accumulation of glycerol under salt stress (Fig. 6e). DsGPDH2/3/5 were upregulated in DsMEK1-X2-oe strains and DsGPDH6 was induced in DsMEK1-X2-RNAi strains under salt stress (Fig. 7). However, the glycerol accumulation rate of DsMEK1-X1 was similar to that of the control lines (Fig. 6e). In addition, the contents of MDA and proline changed in both DsMEK1-X1 and DsMEK1-X2, but the changes were more obvious in DsMEK1-X1 (Fig. 6c, d). MDA and proline were thought to minimize salinity-induced oxidative stress [35]. Based on our results, we speculated that DsMEK1-X1 can mediate various antioxidant systems to reduce oxidative damage and help *D. salina* adapt to salt stress; DsMEK1-X2 is essential for the regulation of glycerol synthesis by activating the expression of DsGPDHs in response to salt stress (Fig. 11). According to previous reports, there are three processes in the salt stress response of *D. salina* [36]. The shrinkage of *D. salina* cells leads to environmental changes, which first lead to osmotic stress followed by oxidation [37]. DsMEK1-X2 participates in the early response through glycerol accumulation, and DsMEK1-X1 participates in the late response to salt stress through antioxidant regulation.

The different functions of DsMEK1-X1 and DsMEK1-X2 are attributed to the change in the regulatory network caused by alternative splicing. We found that there were differences in the interaction networks between DsMEK1-X1 and DsMEK1-X2 through in vitro and in vivo experiments. A yeast two-hybrid assay revealed that DsMEK1 variants displayed a difference in choosing upstream and downstream interactors, DsMAPKKKs and DsMAPK1. DsMEK1-X2 interacted with DsMAPKKK1/2/3/9/10/17 and DsMAPK1; however, DsMEK1-X1 interacted with neither DsMAPKKK1/2/3/9/10/17 nor DsMAPK1 (Fig. 9). Under salt stress, the overexpression strains or knockdown strains of DsMEK1-X2 could change the expression of DsMAPKKK1/2/3/10/17 and DsMAPK1, but the overexpression strains of DsMEK1-X1 did not affect the expression level of these genes, except for DsMAPKKK9 (Fig. 10). Summarizing the present knowledge on DsMEK1-X2, we propose the hypothesis of the MAPK cascade pathway in *D. salina* (Fig. 11). The DsMAPKKK1/2/3/10/17-DsMEK1-X2-DsMAPK1 cascade pathways are essential for the regulation of glycerol synthesis by activating the expression of DsGPDHs in response to salt stress. In Y2H and in vivo overexpression experiments, we did not find the interacting proteins of DsMEK1-X1; therefore, we constructed an overexpression strain of DsMEK1-X1, and a knockdown strain of DsMEK1-X1 was not constructed for further study. The difference between DsMEK1-X1 and DsMEK1-X2 interaction networks may be caused by protein structure. DsMEK1-X1 lacks a part of the NTF2 domain (Fig. 1c), and it has been reported that the knockout of NTF2 will lead to the weakening of protein interaction ability [38]; therefore, we speculate that the difference in the NTF2 domain may lead to the change in DsMEK1-X1 and DsMEK1-X2 regulatory networks. There are two possible reasons for the interaction network of DSMEK1-X1 was not found in this study: 1. In the initial predicted interaction network, because of the high similarity between DsMEK1-X1 and DsMEK1-X2, the results of the interaction network predicted by the STRING database were the same. The interaction network of DsMEK1-X1 may not be affected; 2. DsMEK1-X1 has evolved a MAPK-independent phosphorylation pathway and can directly phosphorylate downstream transcription factors, such as OsMAPK5, directly phosphorylated by CPK18, instead of the MKK-dependent phosphorylation pathway [39]. In this paper, the MAPK cascade pathway of *D. salina* was studied systematically for the first time, which may provide a reference for further research investigating the salt tolerance mechanism of *D. salina*.

## Conclusion

DsMEK1-X1 and DsMEK1-X2 in *D. salina* were successfully cloned and characterized. We showed that overexpression of DsMEK1-X2 enhanced cell growth under salt stress. Overexpression of DsMEK1-X2 in *D. salina* resulted in glycerol accumulation and DsGPDH expression level changes, and the DsMAPKKK1/2/3/10/17-DsMEK1-X2-DsMAPK1 cascade was involved in glycerol synthesis under salt stress (Fig. 11). These results confirmed that a MAPK signalling pathway, similar to the yeast HOG pathway [40], may be involved in the response of *D. salina* to salt stress. Overexpression of DsMEK1-X1 increased the proline content and decreased MDA, and DsMEK1-X1 was induced by H_2_O_2_. These findings revealed that DsMEK1-X1 can reduce the oxidative damage caused by salt stress (Fig. 11). Taken together, the results of this study indicate that AS forms have regulatory functions and may diversify the functions of the DsMEK1 gene in *D. salina*.

## Materials and methods

### Unialgal strains, culture conditions and growth patterns

*D. salina* CCAP (1952-5) was provided by the Freshwater Algae Culture Collection of the Institute of Hydrobiology, Wuhan, China. The culture was maintained in De Walne’s medium and cultivated at 25 °C and 40 μmol m^−2^ s^−1^ provided by cool-white fluorescent lamps, under a 16/8 h light/dark cycle with various NaCl concentrations (1.5 M and 3.5 M) and 0.4 mM H_2_O_2_ [41, 42]. For transformation and selection experiments solid and liquid De Walne’s medium were employed, and adjusted to contain a final concentration of 0.5 M NaCl. For solid medium, 2.5% agar was added [43].

Ten millilitres (7 × 10^6^ cells) of unialgal culture was inoculated in triplicate into 100 mL of De Walne’s culture media and was grown under controlled laboratory conditions to study the growth pattern. Growth was measured in terms of cell numbers using a Neubauer haemocytometer [44].

### Gene cloning, DNA sequencing, bioinformatic analysis, and phylogenetic tree analysis

Through the analysis of the *D. salina* transcriptome and genome data (https://phytozome.jgi.doe.gov/pz/portal.html#!info?alias=Org_Dsalina) [45], one mitogen-activated protein kinase kinase (MAPKK, also known as MEK) gene was found to be highly similar. At the same time, we also used RACE technology to amplify DsMEK1 (Additional file 1: Table S1 No. 1–2) and obtained the complete sequence. Using alignment software to analyse the DsMEK1 sequence cloned by PCR and transcriptome data, we obtained the ORF sequence of DsMEK1. Furthermore, through the analysis of the raw transcriptome data, it was found that there was alternative splicing of this gene, known as DsMEK1-X1 and DsMEK1-X2. The forward and reverse primers used to analyse the CDS of DsMEK1-X1 and DsMEK1-X2 genes (Additional file 1: Table S1 No. 3–4). PCR products were analysed on a 2% agarose gel and then combined with the pMD19-T vector (TaKaRa, China) and target bands were sequenced by Tsingke Co., Ltd. (Beijing, China). The molecular weights (Mw) of the two DsMEK1 splice variants were calculated using the compute Pi/Mw tool online (http://isoelectric.ovh.org/). The identified motifs were annotated based on CDD (https://www.ncbi.nlm.nih.gov/Structure/cdd/wrpsb.cgi). According to the cloned ORF of DsMEK1 and the genome sequence of DsMEK1 in the *D. salina* genome [45], the exon sequence was obtained, and the primers were designed (Additional file 1: Table S2) and amplified by PCR to obtain the complete genome sequence of DsMEK1. The gene structures were analysed with GSDS software (http://gsds.cbi.pku.edu.cn/).

The AtMAPKK sequences were obtained from the Arabidopsis Information Resource (TAIR). The *O. sativa* MAPKK sequences were obtained from the NCBI database. *D. salina* DsMEK2 was obtained from the phytozome database (http://www.phytozome.net/) [46]. In green algae, *C. reinhardtii* CrMAPKK2 and CrMAPKK3, *Ostreococcus lucimarinus* OlMAPKK6, *Volvox carteri* VcMAPKK1 and VcMAPKK2 [47] were obtained from the PLAZA database (http://bioinformatics.psb.ugent.be/plaza/) [48] and phytozome database (Additional file 1: Table S3). Multiple protein sequence alignments were performed using ClustalX 2.0, while a phylogenetic tree was constructed from the amino acid sequences using the neighbour-joining method with MEGA 6 software. A bootstrap analysis was performed using 1000 replicates [49].

### Subcellular localization

For protein localization observation, DsMEK1-X1 and DsMEK1-X2 were inserted into the p1300-GFP expression vector using gene specific primers (primers No. 5–6 in Additional file 1: Table S1) [31]. To transiently express the fusion proteins in protoplasts isolated from Arabidopsis leaves, PEG-mediated transformation was used to transfect the protoplasts with each DsMEK1::GFP construct [50]. GFP was excited by 488 nm laser lines and detected with bandpass 498–543 nm filters.

### Yeast two-hybrid assays

Yeast two-hybrid assays were performed with the Y2H Gold-Gal4 system (Clontech). DsMEK1-X1/2 were inserted into the pGADT7 vectors, and DsMAPK1 (NCBI, GenBank: EF186770.1) and DsMAPKKK genes were inserted into the pGBKT7 vectors to form the bait and prey constructs (primers No. 7–44 in Table S1). The bait and prey constructs were transformed into yeast strain Y2H Gold according to the manufacturer’s instructions (Clontech). The yeast cells were cultured on SD/-Trp/-Leu/-His/-Ade medium containing X-ɑ-gal at 28 °C in the dark for three days.

### Construction of DsMEK1-X1/X2 overexpression strains and DsMEK1-X2 knockdown strains

The pGreen-0029 binary vector was modified and used as the *D. salina* transformation vector. To generate the DsMEK1 transformation constructs, a 573 bp chloramphenicol resistance gene (Cmr) was amplified and inserted into pGreen-0029 as a dominant selectable marker (primers No. 45–46 in Additional file 1: Table S1). To construct pGreen-0029-Cmr-DsMEK1 overexpression strains (DsMEK1-X1-oe and DsMEK1-X2-oe), the full-length CDS of DsMEK1-X1 and DsMEK1-X2 were amplified by PCR (primers No. 47–48 in Additional file 1: Table S1) and then inserted into the modified pGreen-0029-Cmr vector. To generate the DsMEK1-X2 knockdown construct DsMEK1-X2-RNAi strains, interference sequences were obtained by chemical synthesis (Sangon Biotech Co., Ltd) and inserted into the modified pGreen-0029-Cmr vector.

### Transformation of *D. salina*

Constructed plasmids were transformed into *D. salina* cells using the Agrobacterium-mediated transformation method as described previously with slight modifications [51]. The empty vector pGreen-0029-Cmr (control), and constructed plasmids were transformed into *Agrobacterium tumefaciens* GV3101 via the freeze–thaw method.

Approximately 10^7^ cells of *D. salina* were plated onto solid De Walne’s medium. Algae cells were incubated for 1 week until a lawn of cells was formed. Three replicates were maintained for each treatment. The transformed agrobacterium culture was supplemented with 100 μM acetosyringone and incubated for 4 h at 28 °C. *D. salina* cells were then infected using a solid infection medium. For solid infection, 500 μL of induced *A. tumefaciens* was added onto the microalgae culture plate. The treatment plates were further incubated for 2 days at 25 °C in the dark. After co-cultivation, cells were harvested and washed three times. Then, the transformed *D. salina* was plated onto De Walne’s medium agar plates containing 100 mg L^−1^ cefotaxime and 400 mg L^−1^ chloramphenicol as the selection marker. Colonies that appeared within 3 weeks were subcultured in liquid selective medium. Individual DsMEK1-X1-oe, DsMEK1-X2-oe and DsMEK1-X2-RNAi colonies were subjected to genotyping PCR to confirm the presence of gene integration (primers No. 49–50 in Additional file 1: Table S1).

### Total sugar content

The total sugar content of each sample subjected to 3.5 M salinity concentrations was estimated by the procedure of Dubois et al. [44]. Ten millilitres of culture was centrifuged at 5000 rpm for 5 min, and the pellet was resuspended in 4 mL distilled water and incubated at 100 °C for 1 h. Homogenized cells were centrifuged for 10 min at 5000 rpm, and 2 mL of upper aqueous solution was transferred to a fresh test tube. A 1 mL phenol solution (5%) was added to the test tube followed by the addition of 5 mL sulfuric acid. The test tube was kept at room temperature for 30 min. The upper colour phase was removed for the absorbance reading at 485 nm [44]. Reading was compared to determine the total sugar content of samples with a standard curve, which was drawn by the same method, using a known amount of glucose as the standard.

### Proline content

The proline content of each sample subjected to 3.5 M salinity concentrations was measured with a proline measurement kit (Nanjing Jian Cheng Bioengineering Institute, Nanjing, China) according to the manufacturer’s instructions. Each experiment was performed with 3 independent replicates. The proline content was divided by cell number to obtain the proline content per cell.

### MDA content

The MDA content of each sample subjected to 3.5 M salinity concentrations was measured with a malondialdehyde (MDA) assay kit (Nanjing Jian Cheng Bioengineering Institute, Nanjing, China) according to the manufacturer’s instructions. Each experiment was performed with 3 independent replicates. The MDA content was divided by cell number to obtain the MDA content per cell.

### Glycerol content

Intracellular glycerol content of *D. salina* subjected to 3.5 M salinity concentrations was measured using the method of Prabhakar et al. [52] with slight modifications. All samples (5 mL) were centrifuged (6000 g, 5 min at RT), and the pellets were washed with fresh culture medium. The pellets were suspended in distilled water (1 mL). The suspensions were freeze–thaw and then centrifuged (12 000 g, 10 min, RT). The supernatant (200 μL) of each sample was made up to 1 mL by adding distilled water. Then, 1.2 mL sodium periodate reagent was added and mixed followed by the addition of 1.2 mL acetylacetone reagent. These samples were gently mixed and placed in a water bath (60 °C, 10 min) and quickly cooled in an ice bath. The absorbance of each sample was read at 413 nm after cooling at room temperature.

### RNA extraction and qRT-PCR

The total RNA of each sample was isolated with TRIzol™ Reagent (Invitrogen) according to the manufacturer’s instructions. The first cDNAs were synthesized using the ExTaq™ PCR Kit following the manufacturer’s protocol (Takara, China). qRT-PCR was carried out with a BIORAD CF96 Real-Time PCR system using SYBR ^®^ Premix Ex Taq™ II (Takara, China). The primers used for qRT-PCR are listed in Table S4, and the β-tubulin gene was used as an internal reference. Each treatment was repeated three times independently. The 2^−∆∆Ct^ method was used to analyse the relative expression of genes.

## Supplementary information

**Additional file 1: Fig S1.** Full-size sequences of DsMEK1 cDNAs were amplified by rapid amplification of cDNA ends (RACE)-PCR. **Table S1.** Primers and antisense oligonucleotides used in this study. **Table S2.** Primers for DsMEK1 genome amplification. **Table S3.** Nomenclatured gene name locus IDs of plant MAPKKs. **Table S4.** Primer sets used in qRT-PCR.

## Data Availability

All data generated or analyzed during this study are included in this published article and its Additional files.

## Abbreviation

MAPK: Mitogen-activated protein kinase
MAPKK (MEK): Mitogen-activated protein kinase kinase
MAPKKK: Mitogen-activated protein kinase kinase kinase
MDA: Malondialdehyde
Cmr: Chloramphenicol resistance gene
GPDH: Glycerol-3-phosphate dehydrogenase
