# Supplementary material for: Two splice variants of the DsMEK1 mitogen-activated protein kinase kinase (MAPKK) are involved in salt stress regulation in Dunaliella salina in different ways
Source: Biotechnol Biofuels. 2020 Aug 19;13:147. doi: 10.1186/s13068-020-01786-w (PMC7439689; doi:10.1186/s13068-020-01786-w)
Supplement: Supplementary file 1 — Additional file 1: Fig S1. Full-size sequences of DsMEK1 cDNAs were amplified by rapid amplification of cDNA ends (RACE)-PCR. Table S1. Primers and antisense oligonucleotides used in this study. Table S2. Primers for DsMEK1 genome amplification. Table S3. Nomenclatured gene name locus IDs of plant MAPKKs. Table S4. Primer sets used in qRT-PCR. [file 13068_2020_1786_MOESM1_ESM.docx]

Fig S1. Full-size sequences of DsMEK1 cDNAs were amplified by rapid amplification of cDNA ends (RACE)-PCR

>DsMEK1

GTGTTCATAATAAGTCCAAAATATTAAATTGTACTATGTTGCAAAATATCCACGCACTTGTGCTGAACTTGGCTTGCATAACTTAGCTTGCATAACTCATCTTGACCAGGGCACACACAGAGGACCCGGGGATTTAGAGCATCACGATGCCATTGCCCAAGCTACAGCTACAGCTGCCAGTCAATGAATCCCAGAGTGTCAATGAGTCACAGCAGCTGCGAGCATCCATCAATCTCAATGGCTCATTGAGGGCGAACCAGGATGAGGTCACAATGCTGACCCGCTCTTACAATCCGTACAACTTCACCACTGAGGGCTTCACCTCGAAAGGTTCAAACGGAGGGCAACACTACAAGATATCTGAAAAAGATATCTGGATTATAAGGCGGCAGGGCGCAGGCGCCAGCAGCACAGTATTCAAGGGCTTCCATTTCCGTGAGAATCGCTTTGTGGCGGTGAAGAAGATTAATGTGTTGGATCGGGAGACGCGGCACCAAATGCTGAATGACGTCAAGGCGCTCTGCGATGCGCGTGCTGTGCCTGGGCTCATTGCCTTTGCCGGCGCGTTCCACATGCCTGACTCCGGCCAGATTGCCATTGTCCTAGAGTACATGGATGGTGGATCTTTGCAGGATGTGCTGGAGAAGGTGGGTAGCATTCCTGAGGACATCCTGAGCCTCATCACCGCCAGAATATTAGTGGGCCTGACCTACCTGCACCGTCAAAAGCACATGGTGCACAGAGATATAAAGCCTGGCAACATTCTTGTTAATTCAGATGGGGACCCCAAAATCACAGATTTTGGAATCAGCGCATTTATTGACAGCACTCTTGCGGTGTGCAACACGTTCTTGGGCACGGTGACCTACATGTCCCCCGAGCGCATCAACAACGAGCAGTACTCCTTTTCTGCGGACATCTGGTCGTTGGGCTTGGTTCTGATCGAATGCGCAACTGGGAAGTACCCCTACGATGCAAGTGTGGGCCCACTCCAACTCATGATCCAAGTTCTGAATGACGACCTGCCACTACCAGAGGGCCCCAATGTGTCGCCTGAGTTCAAGGACTTCCTCACGCAGTGTCTGCGCAAGAACCCCTATGAGCGCCCCACAGCCGAGCAGCTGCTGCAGCACCCCTTCATCACCAAGCACGCGTCAGGGCCCCCTGGTGCCTTGAAAGCATACATGCACCGAGTATTTGATCCCCATGACAAGCTTGATGAAATCACCATCATCTTCACTTGGAATTACTACGCACTGCTCTCAGGCGGGCAAGGGCAGTTGCAAGCCCTGGCACCCCTGTACTCGGTTGCCTCTGTACTGGAGTATGATGGAGCCAAGTGCAAAGGGCGGCAAGCCATCATGGCGCAGTTGGCGTCAGCATCAGCCTCGCATGCAGCACGCAGGATCCAGAAGCACGAGGTCAAGAGCGTGGACTGCCAGCCTCTGGGCCTGGATGGCAGTGCGCTGGTACATGTGCAAGGCTCACAGGTATCAGAGGTAGCAGGGGATGCTGAAGCAGCTGTGCCCTTCACCGAAGCCTTCATTCTGTGCCAAGTACAGCCAGGAGAGTATTATGTGGCAAACCAAGTGCACCGCACCCTTGCGTAACACTCGTCAGTCTGCCTTCTGATGGAACATTTTTTGGATTTTTTACAACCGTCATAAAAAAAAAAAAAAAAAAAAAAAAAAAAAAAAAAAAGTA

Table S1. Primers and antisense oligonucleotides used in this study

| No. | Name | Sequnce |
| --- | --- | --- |
| 1 | DsMEK1-5’ RACE | 5′-ATCTTCTTCACCGCCACAAAGCGATTCTTC-3′ |
| 2 | DsMEK1-3’ RACE | 5′-AGTTCAAGGACTTCCTCACGCAGTGTCTGC-3′ |
| 3 | DsMEK1_CDS_F | 5′-ATGCCATTGCCCAAGCTACAGCTAC-3′ |
| 4 | DsMEK1_CDS_R | 5′-TTACGCAAGGGTGCGGTGCACTTGG-3′ |
| 5 | DsMEK1-SF | 5′-TCTAGACGATGCCATTGCCCAAGCTACAGCT-3′ |
| 6 | DsMEK1-SR | 5′-GAATTCCGCAAGGGTGCGGTGCACTTGGTT-3′ |
| 7 | DsMAPK1-Y2H-F | 5′-GAATTCATGGCGTCGCGATCCGAGGAGTCCA-3′ |
| 8 | DsMAPK1-Y2H-R | 5′-GGATCCCTAGTTGCCTGAGTGGCTGCCTCTG-3′ |
| 9 | DsMAPKKK1-Y2H-F | 5′-GAATTCATGGATCGCATCGCGGAAGTGCG-3’ |
| 10 | DsMAPKKK1- Y2H-R | 5′-GGATCCTCACCTGCATGGCCCCTTGCTGGCT-3’ |
| 11 | DsMAPKKK2- Y2H-F | 5′-GAATTCATGCTTTTAGGCACCAGCCTCC-3’ |
| 12 | DsMAPKKK2- Y2H-R | 5′-GAGCTCTCGCATCTGGTGCTCACGCGCG-3’ |
| 13 | DsMAPKKK3- Y2H-F | 5′-GAATTCATGTGGGAAGCTTGGCGAGAGCA-3’ |
| 14 | DsMAPKKK3- Y2H-R | 5′-GGATCCTCACTTGCTGAGACCGAAGTCACAA-3’ |
| 15 | DsMAPKKK4- Y2H-F | 5′-GAATTCATGTTCCCCATGAACGATGAGCGTG-3’ |
| 16 | DsMAPKKK4- Y2H-R | 5′-GGATCCTCAGGACACCGTGCAGCATTCCTGG-3’ |
| 17 | DsMAPKKK5- Y2H-F | 5′-GAATTCATGACCCTGCAAAGCCTGCGGCACC-3’ |
| 18 | DsMAPKKK5- Y2H-R | 5′-GGATCCTCACAATTGCTTTGCTACCTTAGAG-3’ |
| 19 | DsMAPKKK6- Y2H-F | 5′-GAATTCATGGACGAGCATGGCATGCTGAACC-3’ |
| 20 | DsMAPKKK6- Y2H-R | 5′-GGATCCTACCTCAAAGCGGACCCCCCCGGAC-3’ |
| 21 | DsMAPKKK7- Y2H-F | 5′-GAATTCATGGGTAGCAGTCCGTCCAAGACCT-3’ |
| 22 | DsMAPKKK7- Y2H-R | 5′-GAGCTCGAAGGTGCCCACCAGCTCATCC-3’ |
| 23 | DsMAPKKK8- Y2H-F | 5′-GGATCCATGCTGGTGTCTCAGCCCTCGCAGT-3’ |
| 24 | DsMAPKKK8- Y2H-R | 5′-CTCGAGCTATTTATGGCCGCGCAGCTTGGCC-3’ |
| 25 | DsMAPKKK9- Y2H-F | 5′-GAATTCATGTCGCGTGCCCCCCCAGCCTC-3’ |
| 26 | DsMAPKKK9- Y2H-R | 5′-GGATCCTCACTGCAACGAGCAGCACTCCCTG-3’ |
| 27 | DsMAPKKK10- Y2H-F | 5′-GAATTCATGGTGGCCTTGGTTGGGGCACCCA-3’ |
| 28 | DsMAPKKK10- Y2H-R | 5′-GGATCCTTACCTCTCCTTGTCACCAGATCGT-3’ |
| 29 | DsMAPKKK11- Y2H-F | 5′-GAATTCATGGGGGGCGGCTTGGACCTGCC-3’ |
| 30 | DsMAPKKK11- Y2H-R | 5′-GAGCTCTCACACCCTGACCAGCTTCCCCAAC-3’ |
| 31 | DsMAPKKK12- Y2H-F | 5′-GAATTCATGGTGGCGCAGGTAGAGCGGGCAT-3’ |
| 32 | DsMAPKKK12- Y2H-R | 5′-GAGCTCTCTGCTTGCTAACCAGGGGTGCTGG-3’ |
| 33 | DsMAPKKK13- Y2H-F | 5′-GAATTCATGGGGGGTTGTTTCTCAAGCAC-3’ |
| 34 | DsMAPKKK13- Y2H-R | 5′-GGATCCTCAGTGTAATGATTGCTCCGAACCT-3’ |
| 35 | DsMAPKKK14- Y2H-F | 5′-GAATTCATGGGCCCAGGCCAACCTCAATTCC-3’ |
| 36 | DsMAPKKK14- Y2H-R | 5′-GAGCTCTCACGCAGGCAGTTTCAACAAAGAC-3’ |
| 37 | DsMAPKKK15- Y2H-F | 5′-GAATTCATGGGTGCATGCGTGAGCAAAGACA-3’ |
| 38 | DsMAPKKK15- Y2H-R | 5′-GGATCCGCTGGGGGGTGGGGTTGCAGGGTCT-3’ |
| 39 | DsMAPKKK16- Y2H-F | 5′-GAATTCATGGGTCTGTTCTCATGCTTCGCC-3’ |
| 40 | DsMAPKKK16-Y2H-R | 5′-GGATCCGAAGTACAACTCGCTGGGCGGCTGC-3’ |
| 41 | DsMAPKKK17-Y2H-F | 5′-GAATTCATGGGCTGTTTCGGGGGTTCATCTA-3’ |
| 42 | DsMAPKKK17-Y2H-R | 5′-GGATCCACCTTGACCCGCACCCCCTCCTCCT-3’ |
| 43 | DsMEK1-Y2H-F | 5′-GAATTCATGCCATTGCCCAAGCTACAG-3’ |
| 44 | DsMEK1-Y2H-R | 5′-GAGCTCTTACGCAAGGGTGCGGTGCA-3’ |
| 45 | Cmr-F | 5′-GTCGAC TTGATCGGCACGTAAGAGGTTCCAA-3′ |
| 46 | Cmr-R | 5′-AAGCTTAATTTCTGCCATTCATCCGCTTATT-3′ |
| 47 | DsMEK1-oe-F | 5’-TCTAGAATGCCATTGCCCAAGCTACAGCTAC-3’) |
| 48 | DsMEK1-oe-R | 5’-GAGCTCTTACGCAAGGGTGCGGTGCACTTGG-3’ |
| 49 | verify-Cmr-F | 5’-CTCTTCAGCAATATCACGGGTAGC-3’ |
| 50 | verify-Cmr-R | 5’- TGCGTATAATATTTGCCCATGGTGA-3’ |

The red character represents the enzyme cleavage site.

Table S2. Primers for DsMEK1 genome amplification

| Name | Sequence of primer（5’-3’） |
| --- | --- |
| DsMEK1-G-F1 | ATGCCATTGCCCAAGCTACAGC |
| DsMEK1-G-R1 | CTCTAGGACAATGGCAATCTGG |
| DsMEK1-G-F2 | ATCCTGCACTCGCTATTGCAC |
| DsMEK1-G-R2 | ATATTCTGGCGGTGATGAGGCTC |
| DsMEK1-G-F3 | AACAGCAGTTCTGAGGGCAACCA |
| DsMEK1-G-R3 | ATGTAGGTCACCGTGCCCAAGAA |
| DsMEK1-G-F4 | ACATAGATTGTGCAGTGTGCCA |
| DsMEK1-G-R4 | AGTCCTTGAACTCAGGCGACACAT |
| DsMEK1-G-F5 | AGCTCATGGCTTCCTCACGCA |
| DsMEK1-G-R5 | GCACTTGGCTCCATCATACTCC |
| DsMEK1-G-F6 | ATGCTTCTGTCTGTGGGCCCTG |
| DsMEK1-G-R6 | ACGCTCTTGACCTCGTGCTTCT |
| DsMEK1-G-F7 | ATGCACGCGCATGTGCATGCAGTCC |
| DsMEK1-G-R7 | TTACGCAAGGGTGCGGTGC |

Table S3. Nomenclatured gene name locus IDs of plant MAPKKs

| Gene name | Locus ID | ORF | No. of a.a |
| --- | --- | --- | --- |
| Arabidopsis thaliana | | | |
| AtMAPKK1 | At4G26070 | 1065 | 335 |
| AtMAPKK2 | At4G29810 | 1092 | 364 |
| AtMAPKK3 | At5G40440 | 1563 | 521 |
| AtMAPKK4 | At1G51660 | 1101 | 367 |
| AtMAPKK5 | At3G21220 | 1047 | 349 |
| AtMAPKK6 | At5G56580 | 1071 | 357 |
| AtMAPKK7 | At1G18350 | 924 | 308 |
| AtMAPKK8 | At3G06230 | 882 | 294 |
| AtMAPKK9 | At1G73500 | 933 | 311 |
| AtMAPKK10 | At1G32320 | 918 | 306 |
| *Chlamydomonas reinhardtii* | | | |
| CrMAPKK2 | CR13G00450 | 1422 | 474 |
| CrMAPKK3 | CR06G00090 | 1359 | 453 |
| *Dunaliella salina* | | | |
| DsMEK1-X1 | 20026_g1 | 1017 | 339 |
| DsMEK1-X2 | 20026_g1 | 1461 | 487 |
| DsMEK2 | Dusal.0024s00003.1 | 1185 | 395 |
| *Oryza sativa* | | | |
| OsMAPKK1 | Os06G05520 | 1059 | 353 |
| OsMAPKK2 | Os06G27890 | 1572 | 524 |
| OsMAPKK3 | Os02G54600 | 1110 | 370 |
| OsMAPKK4 | Os06G09180 | 1029 | 343 |
| OsMAPKK5 | Os01G32660 | 1068 | 356 |
| OsMAPKK6 | Os02G46740 | 1023 | 341 |
| OsMAPKK9 | Os03G12390 | 1020 | 340 |
| OsMAPKK10 | Os03G50550 | 1038 | 346 |
| *Ostreococcus lucinarinus* | | | |
| OlMAPKK6 | OL04G03760 | 801 | 267 |
| *Volvox carteri* | | | |
| VcMAPKK1 | Vocar.0038s0010.1 | 1056 | 352 |
| VcMAPKK2 | Vocar.0028s0061.1 | 1368 | 456 |

Table S4. List of primer sets used in qRT-PCR

| Gene name | Primer name | Primer sequences |
| --- | --- | --- |
| β-tubulin | β-tubulin-F  β-tubulin-R | 5′ GTGGAGAACGCCGATGAGT 3′  5′ AGCAGGTGACACCGGACAT 3′ |
| DsMEK1-X1 | DsMEK1-X1-F  DsMEK1-X1-R | 5′ ACTTTGCGTTGCAAGCCCT3′  5′ CTGGCAGTCCACGCTCTTGA 3′ |
| DsMEK1-X2 | DsMEK1-X2-F  DsMEK1-X2-R | 5′ CCTGGTGCCTTGAAAGCATACA 3′  5′ CTGGCAGTCCACGCTCTTGA 3′ |
| DsGPDH1 | DsGPDH1-F  DsGPDH1-R | 5′ TTTTGAGAGTTGTGGTTTGGGAGA 3′  5′ TGTAGAGAACAGGGGATAGGTG 3′ |
| DsGPDH2 | DsGPDH2-F  DsGPDH2-R | 5′ GTAAAGCCTCCATCCTGCGAC 3′  5′ GCCGTTCAGCATCTCCTTCTC 3′ |
| DsGPDH3 | DsGPDH3-F  DsGPDH3-R | 5′ CTGCGGACTGTGTGTGCTTTGA 3′  5′ GCCTCGTTTCTGTAGCGTCTTGA 3′ |
| DsGPDH4 | DsGPDH4-F  DsGPDH4-R | 5′ ATGAGGAGAAGCACAGCGGA 3′  5′ AGCCAACTGCTTACAGATGCC 3′ |
| DsGPDH5 | DsGPDH5-F  DsGPDH5-R | 5′ TTGCTATGGCAGCCTTTCTC 3′  5′ GATTTGTGCTATCCCCGTGA 3′ |
| DsGPDH6 | DsGPDH6-F  DsGPDH6-R | 5′ GCACCTCAACCCTTGTTCCTTC 3′  5′ AACACCTTGCCTTGGTCATCC 3′ |
| DsGPDH7 | DsGPDH7-F  DsGPDH7-R | 5′ ATGTGGACACCGAGATTGCTG 3′  5′ GCCAAAATCTCTGAAATGCGT 3′ |
| DsMAPK1 | DsMAPK1-F  DsMAPK1-R | 5′ AAGACATGGCTGCGCTTCACC 3′  5′ AAGCCCGTCAGTCTTCTCCTCT 3′ |
| DsMAPKKK1 | DsMAPKKK1-F  DsMAPKKK1-R | 5′ GATGGTGGTGTGGGAGGCT 3′  5′ CGGACTCCTTCATCGGCTT 3′ |
| DsMAPKKK2 | DsMAPKKK2-F  DsMAPKKK2-R | 5′ CTGGTAGACAGGGACTGGACFG 3′  5′ TGAGTAGCCGCTTCTTGTTGA 3′ |
| DsMAPKKK3 | DsMAPKKK3-F  DsMAPKKK3-R | 5′ GGCTCAAACCCCACTACCTT 3′  5′ CTCCTGCTCCACACAGATACG 3′ |
| DsMAPKKK9 | DsMAPKKK9-F  DsMAPKKK9-R | 5′ AGACACCAGGTTGCCTTCCA 3′  5′ CCAGAAGGGAGGCACACACT 3′ |
| DsMAPKKK10 | DsMAPKKK10-F  DsMAPKKK10-R | 5′ TGCTTGGAGAGGGAGGGTTC 3′  5′ CGTTCTTCTCGTTCTGTCCG 3′ |
| DsMAPKKK17 | DsMAPKKK17-F  DsMAPKKK17-R | 5′ CAACGAGCAGACGCAGCAA 3′  5′ CGCAACAGGTGGTGGATGA 3′ |
